# Noniterative Fermi–Löwdin Orbitals for Self-Interaction Correction

**DOI:** 10.1021/acs.jpca.6c00022

**Published:** 2026-02-20

**Authors:** Juan E. Peralta, Koblar A. Jackson, Mark R. Pederson, Juan I. Melo, Diego R. Alcoba, Gustavo E. Massaccesi, Luis Lain, Alicia Torre, Ofelia B. Oña

**Affiliations:** † Department of Physics, 5649 Central Michigan University, Mount Pleasant, Michigan 48859, United States; ‡ Department of Physics, 12337 The University of Texas at El Paso, 500 West University Avenue, El Paso, Texas 79968, United States; § 28196 Universidad de Buenos Aires, Facultad de Ciencias Exactas Y Naturales, Departamento de Física, Ciudad Universitaria, 1428 Buenos Aires, Argentina; ∥ CONICET - Universidad de Buenos Aires, Instituto de Física de Buenos Aires (IFIBA), Ciudad Universitaria, Buenos Aires 1428, Argentina; ⊥ Departamento de Ciencias Exactas, Ciclo Básico Común, Universidad de Buenos Aires, Ciudad Universitaria, 1428 Buenos Aires, Argentina; # Instituto de Investigaciones Matemáticas “Luis A. Santaló” (IMAS), Consejo Nacional de Investigaciones Científicas Y Técnicas, Universidad de Buenos Aires, ​Ciudad Universitaria, 1428 Buenos Aires, Argentina; ¶ Department of Physical Chemistry, Faculty of Science and Technology, University of the Basque Country. P.O. Box 644, Bilbao E-48080, Spain; ∇ Instituto de Investigaciones Fisicoquímicas Teóricas y Aplicadas, Universidad Nacional de La Plata, Consejo Nacional de Investigaciones Científicas y Técnicas, Diag. 113 y 64 (S/N), Sucursal 4, CC 16, 1900 La Plata, Argentina

## Abstract

We introduce the noniterative Fermi–Löwdin orbital self-interaction correction (NIFLOSIC) method as a computationally efficient alternative to traditional Fermi–Löwdin orbital self-interaction correction (FLOSIC) by eliminating the need for iterative relaxation of Fermi orbital descriptors (FODs). This is accomplished using the selected columns of the density matrix localization scheme [*J. Chem. Theory Comput.* **2023,** *19,* 8572] and by exploiting the relationship between the electron localization function and FODs [*J. Chem. Phys.* **2025,** *162,* 144105]. The approach produces localized orbitals that are slightly more compact than grid-based selected columns of the density matrix orbitals and generates FODs in a single, noniterative self-starting step, following density functional theory calculations. Within a generalized Kohn–Sham framework, full relaxation of the density minimizes the Perdew–Zunger energy functional, yielding self-interaction corrected densities and orbitals. NIFLOSIC reproduces results from fully self-consistent FLOSIC calculations, while significantly reducing computational cost. Although the total electronic energy is not suitable for thermochemistry, benchmark tests across diverse molecular systems demonstrate that NIFLOSIC significantly improves frontier molecular orbital energies and dipole moments, establishing a practical and scalable approach for large-scale electronic structure applications where self-interaction correction is needed.

## Graphical abstract

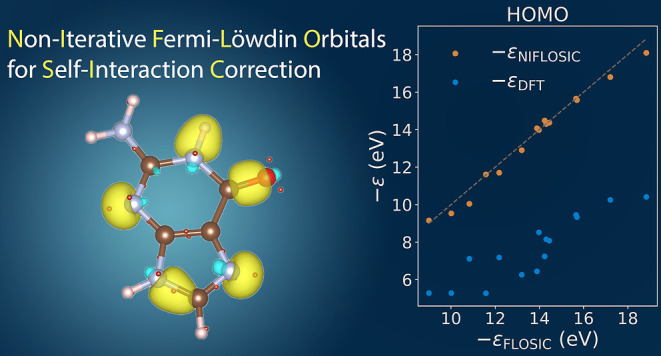

## Introduction

Density functional theory (DFT)^1,2^ has become a cornerstone in electronic structure calculations, largely due to its effective balance between computational cost and accuracy.^3,4^ However, a significant limitation of approximate exchange–correlation (XC) functionals is their failure to eliminate the self-interaction (SI) of electrons, leading to the well-known self-interaction error (SIE). This error underlies several recognized shortcomings of lower-rung density-functional approximations, such as producing incorrect dissociation curves for asymmetric molecules,^5,6^ or predicting overly high energies for the highest occupied molecular orbitals (HOMO) in negatively charged species, which results in an artificially positive charge.^7^

In the early 1970s, Lindgren^8^ proposed correcting HOMO levels by modifying the local density approximation (LDA) Hamiltonian with a self-Coulomb interaction. In 1981, Perdew and Zunger introduced a method to eliminate the one-electron SIE on an orbital-by-orbital basis.^9^ Commonly referred to as the Perdew–Zunger (PZ) self-interaction correction (SIC), this approach modifies the energy functional to depend explicitly on individual orbitals

1 $$E_{PZ‐SIC}=E_{DFT}\left[n^{↑},n^{↓}\right]-\sum_{i,\sigma }\left(E_{XC}\left[n_{i}^{\sigma },0\right]+E_{H}\left[n_{i}^{\sigma }\right]\right)$$

In eq 1, *n*_*i*_^σ^ denotes the spin density of a single orbital (σ = ↑, ↓), while *E*_XC_ and *E*_H_ are the exchange–correlation and Hartree energies, respectively. Minimizing this functional results in localized orbitals. Despite its conceptual appeal, the PZ-SIC method has not seen widespread use in routine calculations, in part due to the computational expense of minimizing the energy, which requires variations of the individual orbital densities in *E*_PZ‑SIC_. Moreover, applying PZ-SIC alongside conventional approximate functionals often degrades the accuracy of various properties. This challenge has motivated the development of DFT approximations compatible with SIC.^10–16^

Several strategies have been developed to minimize *E*_PZ‑SIC_, many of which involve fully relaxing the (localized) orbitals using diverse techniques. These approaches differ primarily in how they handle the additional variational parameters required to describe localized orbitals. Some methods enforce localization conditions directly through localization equations,^17–21^ while others adopt alternative strategies such as the constrained gradient search proposed by Vydrov and Scuseria,^22^ the two-step unitary optimization method introduced by Lehtola and Jónsson,^23,24^ or the Koopmans-compliant functional framework developed by Ferretti et al.^25^ Additionally, several implementations utilize variants of the optimized effective potential (OEP) method.^26–28^

An alternative method for minimizing *E*_PZ‑SIC_ involves the use of Fermi–Löwdin orbitals (FLOs).^29,30^ This technique, known as FLOSIC, is based on the construction of localized orbitals by parametrizing them as Fermi orbitals^31^

2 $$f_{a}\left(r\right)=\frac{n\left(a,r\right)}{\sqrt{n\left(a,a\right)}}$$

where **a** denotes spatial points referred to as Fermi orbital descriptors (FODs), and *n*(**r**′, **r**) is the density matrix (with spin indices omitted for clarity). Originally proposed by Luken et al.^32,33^ to build nonorthogonal localized orbitals, this approach takes advantage of the real-space decay of the density matrix in systems with a finite energy gap,^34,35^ and identifies the rows of a scaled density matrix as localized orbitals. The nonorthogonal Fermi orbitals *f*_*a*_(**r**) are orthogonalized using the Löwdin scheme,^36^ resulting in Fermi–Löwdin orthonormal orbitals. The denominator in $1/\sqrt{n\left(a,a\right)}=1/\sqrt{n\left(a\right)}$ in eq 2 ensures a balanced weighting during symmetric orthogonalization by normalizing the Fermi orbital. Since FLOSIC introduces FODs as additional variational parameters, minimizing *E*_PZ‑SIC_ requires full relaxation of both the electron density and all FODs. Early implementations of FLOSIC employed Jacobi (or Givens^37^)-type rotations to eliminate the overlap between occupied and virtual orbitals at each self-consistent iteration.^38^ More recently, a mean-field approach for density relaxation within FLOSIC has been proposed.^39^ Other implementations based on unified Hamiltonian frameworks and effective potentials have also emerged.^40–44^ In these methods, FODs are relaxed between self-consistent field cycles in a double-loop fashion, keeping the density fixed during each FOD update. Alternatively, one can reformulate the problem as a FOD minimization where each evaluation of the energy and its gradients is performed after self-consistency. Importantly, it has been shown that given a set of FODs, and due to the localized nature of the FLOs, the cost of a FLOSIC calculation can scale similarly to that of DFT in the large system regime.^7^ Thus, methods that allow the derivation of FODs directly from inexpensive evaluations of the Kohn–Sham (KS) orbitals, or alternatively density matrices, would be highly desirable.

A class of orbital localization methods that explicitly avoid demanding iterative minimization schemes has been proposed.^45–47^ In particular, the selected columns of the density matrix grid-based (SCDM-g) method provides a noniterative approach to orbital localization by selecting columns of the density matrix evaluated on a real-space grid,^47^ much like the original idea of Luken and Beratan^32^ The SCDM-g localization technique, which constructs nonorthogonal localized molecular orbitals using a QR decomposition, is independent of predefined atomic orbitals, and its performance is notably robust with respect to grid resolution and basis set contraction schemes. The nonorthogonal localized orbitals can be seen as the density matrix columns that minimize the overlap matrix condition number. These orbitals are then symmetrically orthogonalized to give the set of orthonormal localized molecular orbitals (LMOs).

Using the locality of the density matrix in a local basis set representation, we have introduced a method called DOCSIC (for density matrix as orbital coefficients SIC),^48^ as a simplification of the FLOSIC method. DOCSIC utilizes columns of the orbital representation of the density matrix to construct localized orbitals in PZ-SIC calculations, avoiding the need to incorporate additional variational parameters. This approach enabled a self-consistent generalized KS implementation of SIC and offers a fast alternative to traditional self-interaction correction schemes for cases where SIE is dominant, such as those in the SIE 4 × 4 test set of Grimme et al.^49^ for binding energies. However, DOCSIC provides only moderately localized orbitals and corrects HOMO and LUMO energies only slightly with respect to DFT.

In this work we introduce a new approach to FLOSIC calculations that utilizes FODs and constructs FLOs based on a variation of the SCDM-g method. The method can be used to obtain localized orbitals, determine FODs for FLOSIC calculations, and allows for relaxing the density matrix to minimize the PZ energy functional.

## Theory and Implementation

The FLOSIC approach generates localized orbitals by utilizing Fermi orbitals, as defined in eq 2. These are subsequently symmetrically orthonormalized, resulting in FLOs. Within the PZ-SIC framework, these orbitals are used to remove SIE on an orbital-by-orbital basis. As mentioned in the Introduction, a key ingredient in the FLOSIC approach is the determination of the set of FODs needed to construct these Fermi orbitals. In practice, for a given molecular system, an initial set of FODs is determined (either empirically or from existing data) and then iteratively relaxed to minimize the PZ energy functional. In a recent work, some of us have shown a formal link between FODs and the electron localization function (ELF).^50^ Specifically, ELF values equal to one necessarily imply stationary FODs, while ELF critical points empirically resemble the FOD structure obtained from FLOSIC calculations.

Through a rank-revealing QR factorization, the original SCDM-g method constructs the most linearly independent orbitals, favoring points located where the electron density is significant (or equivalently, where the MOs have a significant value).^47^ Using the connection between the ELF and FODs, here we introduce a methodology based on a modified SCDM-g localization scheme^46,47^ to determine a set of FODs for FLOSIC calculations in a self-starting, noniterative fashion. To this end, we normalize the MO values and multiply by the ELF at each grid point, aiming to favor the selection of points where the ELF is maximal, which is a desirable feature for the FLOSIC method as discussed before.^50^ This scheme balances the ability of the rank-revealing QR factorization to find most linearly independent Fermi orbitals and maximal ELF points at the same time. The process is as follows:

1. From a DFT calculation, evaluate all the *N* molecular orbitals (MO) {ψ_*b*_} (*N*_occ_ occupied and *N*_virt_ virtual), the density *n*(**r**), and the ELF, *F*_ELF_(**r**) on all the *K* points of a quadrature grid {**r**_*j*_} (the standard integration or a coarser grid) and evaluate the *N* × *K* matrix $g_{b}\left(r_{j}\right)=\psi_{b}\left(r_{j}\right)/\sqrt{n\left(r_{j}\right)}$ . Normalize this quantity to obtain $0\leq {\mathrm{g̃}}_{b}\left(r_{j}\right)\leq 1$ . Evaluate $A_{bj}={\mathrm{g̃}}_{b}\left(r_{j}\right)\times F_{ELF}\left(r_{j}\right)$ on the grid.
2. Perform a rank-revealing QR factorization with pivoting on the rectangular *N* × *K* matrix **A** of elements *A*_*bj*_. This QR factorization with pivoting provides a solution to **AΠ** = **QR**, where **Q** and **R** are orthonormal and upper-triangular matrices, respectively, and **Π** is a permutation matrix. **Π** is chosen so that the diagonal elements of **R** are nonincreasing, i.e. |*R*_*ii*_| ≥ |*R*_*i*+1 *i*+1_|. Keep only the first *N*_occ_ elements of the permutation matrix, **Π**_occ_.
3. The grid points {**a** | **r**_*j*_ ∈ **Π**_occ_} are the FODs to be used to construct Fermi orbitals,

3 $$f_{a}\left(r\right)=\sum_{b\in occ}\frac{\psi_{b}^{*}\left(a\right)\psi_{b}\left(r\right)}{\sqrt{n\left(a\right)}}$$

The FLO coefficient matrix **X** in the atomic orbital (AO) basis {ϕ_μ_} can be evaluated as **X** = **GO** ^1/2^, where **G** = **PY**, **O** = **Y** ^†^ **PY** is the Fermi orbitals overlap matrix, and the matrix **Y** has elements $Y_{\mu a}=\phi_{\mu }\left(a\right)/\sqrt{n\left(a\right)}$ . The single-orbital density matrices **P**_*a*_ can be evaluated from the elements of **X** as ${\left[P_{a}\right]}_{\mu \nu }=X_{a\mu }^{†}X_{a\nu }$ .

The resulting noniterative Fermi–Löwdin orbital (NIFLO) single-orbital coefficient matrix **X** and density matrices **P**_*a*_ can be used to evaluate the orbitals and their corresponding densities, while the grid points **a** can be used as FODs for FLOSIC calculations. The effective generalized KS Hamiltonian can be obtained as

4 $$H_{PZ‐SIC}\equiv \frac{dE_{PZ‐SIC}}{dP}=\frac{dE_{DFT}}{dP}+\frac{dE_{SIC}}{dP}$$

where the first term in the r.h.s of eq 4 is the standard KS Hamiltonian, and *E*_SIC_ refers to the second term on the r.h.s of eq 1. This derivative can be written as

5 $$\frac{dE_{SIC}}{dP}\equiv H_{SIC}=\sum_{a}\frac{dE_{SIC}}{dP_{a}}\cdot \frac{dP_{a}}{dP}$$

We have implemented the NIFLO approach using an in-house modification of the PySCF electronic structure code.^51^ The derivatives in eq 5 were evaluated using the automatic differentiation Autograd from Torch.^52^ We note that, alternatively, one can obtain **H**_**SIC**_ using the procedure outlined in ref 39. The current implementation assumes real-valued density matrices and hence leads to real-valued localized orbitals. With the generalized multiplicative Hamiltonian matrix in eq 4, the self-consistent procedure can be performed as in any standard DFT calculation. It should be emphasized that this procedure employs the grid points {**a**} utilized to generate the localization transformation from orbitals obtained from a DFT (not SIC) calculation. However, since this transformation depends also on the density matrix, the transformation itself is updated at every iteration. We refer to this approach as NIFLO self-interaction correction, or NIFLOSIC. A version of the code, including the initial FOD generator, NIFLO localized orbitals, and NIFLOSIC and standard FLOSIC calculations, is publicly available in https://github.com/peraltajuan/NIFLO.

## Results

All calculations in this work utilize the density functional approximation of Perdew, Burke, and Ernzerhof (PBE)^53,54^ with the def2-TZVPD basis set.^55,56^ We first assess the locality of the resulting NIFLOs by comparing the total orbital variance $\sigma =\sqrt{\sum_{i}{\left\langle r-{\left\langle r\right\rangle }_{i}^{2}\right\rangle }_{i}}$ with the SCDM-g and Foster–Boys (FB) orbitals. The latter minimize the mean extension of the orbitals from their centroids, and thus have the minimum possible orbital variance. In Figure 1, we show this comparison for a set 676 randomly selected molecules from the GDB-11 database containing from 3 to 11 first-row elements.^57^ The plot shows that the NIFLO method yields localized orbitals that are more compact than their SCDM-g counterparts. Both localized orbitals are significantly more localized than noniterative Cholesky orbitals (not shown in Figure 1).^45^

**Figure 1:**
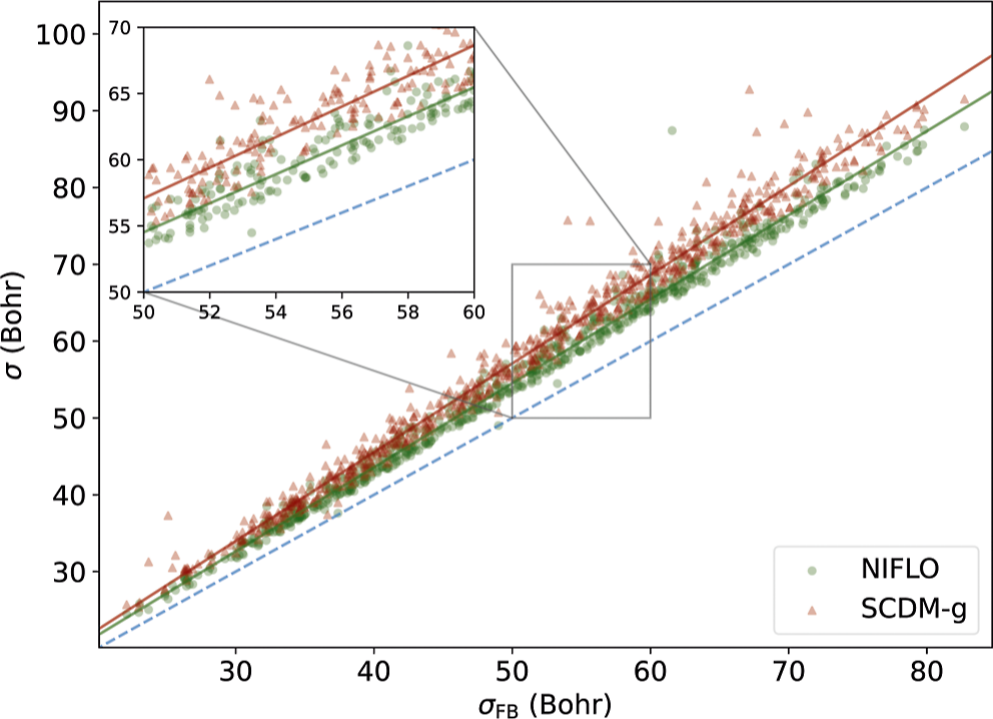
Total localized orbital variance, σ, for a set of 676 molecules. The abscissa corresponds to Foster–Boys (FB) orbitals (minimum possible variance) while the ordinate shows the noniterative Fermi–Löwdin orbitals (NIFLO) and selected columns of the density matrix, grid version (SCDM-g) orbitals. The inset shows a zoom in the central region of the plot. The red and blue lines show the best linear fit for each class of localized orbitals. The 1:1 line is shown in dashes.

SIE leads to an artificial electron delocalization in functionals of LDA and generalized gradient approximation (GGA) families, which systematically raises the energy of the HOMO and lowers the energy of the lowest unoccupied molecular orbital (LUMO). This results in a well-known unphysical underestimation of the HOMO–LUMO gap in molecules and band gap in solids, often associated with the delocalization error in DFT.^58–62^ Thus, it makes sense to assess the ability of the NIFLOSIC method to reproduce frontier orbital energies as calculated by FLOSIC. To this end, we compare the HOMO and LUMO energies for a diverse set of molecules using standard DFT, the NIFLOSIC, and fully self-consistent FLOSIC approaches. Figure 2 summarizes the results for 17 representative molecules (structures taken from ref 63), ranging from simple hydrides to more complex systems. The HOMO energies predicted by conventional DFT are significantly underestimated with respect to FLOSIC, as it is well-known, with a mean absolute deviation (MAD) of 5.90 eV with respect to the FLOSIC energies. NIFLOSIC HOMO energies, in contrast, align much better with FLOSIC HOMO energies, with a MAD of 0.30 eV. A statistical summary of these results is shown in Table 1. The effect of removing SIE in LUMO energies is less pronounced, as shown in Figure 2 and this is reflected on both the NIFLOSIC and FLOSIC methods.

**Figure 2:**
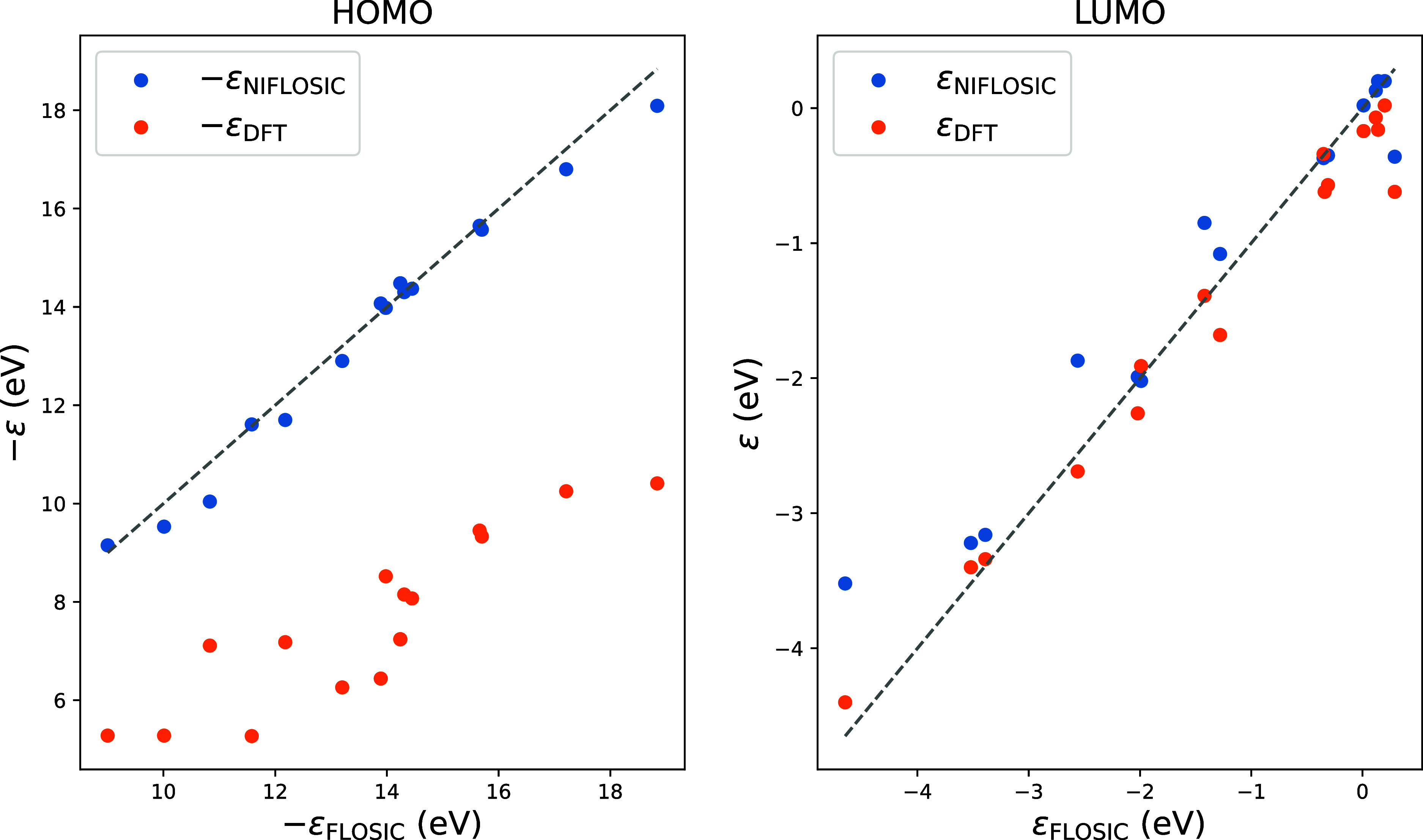
HOMO (left panel) and LUMO (right panel) energies in eV for each method. The set of molecules is composed of CH_4_, H_2_O, SiH_4_, CO, CO_2_, SO_2_, N_2_, P_2_, NaCl, C_2_H_6_, CF_4_, H_2_NNH_2_, HOOH, H_2_CO, C_2_H_2_, and C_5_H_5_N_5_O. Dashed lines show the 1:1 agreement.

**Table 1:** HOMO and LUMO Mean Absolute Deviation (MAD) and Mean Deviation (MD) with Respect to FLOSIC Values for the Data Shown in Figure 2

	HOMO		LUMO	
molecule	DFT	NIFLOSIC	DFT	NIFLOSIC
MAD	**5.85**	**0.32**	**0.24**	**0.24**
MD	**5.85**	**0.15**	**–0.18**	**0.12**

SIE causes spurious charge transfer within a molecule and results in inaccurate predictions of the molecular dipole moment. Correcting for SIE generally increases the degree of charge transfer, thus increasing the size of calculated dipole moments.^64^ In Table 2 we show the molecular dipole moments calculated for the 8 nonzero dipole cases in Table 1. NIFLOSIC corrects the DFT dipole moment toward the FLOSIC value in all cases, indicating that the resulting density from the self-consistent NIFLOSIC calculation effectively captures the self-interaction correction.

**Table 2:** Dipole Moments (in Debye) Calculated Using DFT, NIFLOSIC, and FLOSIC Methods

molecule	DFT	NIFLOSIC	FLOSIC
H_2_O	1.80	1.87	1.93
CO	0.29	0.89	0.69
SO_2_	1.48	1.89	1.79
NaCl	8.53	8.99	9.00
H_2_NNH_2_	2.63	2.79	2.83
HOOH	1.52	1.57	1.61
H_2_CO	2.23	2.96	2.61
C_5_H_5_N_5_O	6.55	6.88	6.73

The results in Table 1 showcase the potential of the NIFLOSIC method for performing self-interaction corrected band structure calculations in solids at a fraction of the cost of full FLOSIC calculations. As a reference, for the set of molecules analyzed in this work, NIFLOSIC calculations take on average about 13 times more than DFT calculations, while the full FLOSIC calculations take about 1300 times that (as shown in Table 3). This large difference is mainly due to the additional time required to fully relax the FODs in FLOSIC calculations, while in NIFLOSIC FODs are determined noniteratively only once.

**Table 3:** Summary of FLOSIC and NIFLOSIC Calculation Details^a^

	NIFLOSIC	FLOSIC
*t*/*t*_DFT_	13	1326
|*g*| (mE_*h*_/Bohr)	3.84	0.07
Δ*E* (mE_*h*_)	-	–26.7
Δ*R* (Bohr)	-	0.17

a FLOSIC calculations use the results of the NIFLOSIC calculations as starting point. |*g*| is the average FOD gradient norm per FOD at the end of the calculations, Δ*E* is the average energy change in the FLOSIC calculations relative to NIFLOSIC, Δ*R* is the average absolute FOD displacement per FOD during the FLOSIC optimization, and *t*/*t*_DFT_ is the average computational time normalized to standard DFT calculations.

In this work, the grid points {**a**} determined for the construction of the NIFLOs are subsequently employed as initial positions for the FODs, while the converged NIFLOSIC density serves as the initial guess for the FLOSIC calculations, underscoring a practical applicability of the method. This allows us also to inspect the behavior of key quantities upon relaxing FODs. Table 3 shows the average energy decrease for the set of 17 molecules in Table 1. One immediate observation from this is that the NIFLOSIC total energies are close to the fully relaxed FLOSIC energies, but not sufficiently close to perform general thermochemistry (the gain in energy is on average ∼17 kcal/mol or 0.73 eV), except in cases where SIE is dominant^48^ or potentially with SIC schemes where the total energy is relatively insensitive to the FOD positions. The average displacement per FOD in the self-consistent process is ∼0.17 Bohr and the FOD gradient norm for each FOD decreases in average from 3.84 to 0.07 mE_*h*_/Bohr. The NIFLOSIC method provides a fast nonempirical self-starting set of orbitals and FODs for FLOSIC calculations, at a cost that is on average about 12 times the cost of a DFT calculation for the small molecules considered here. In contrast, the full FLOSIC optimization computational cost using a tight tolerance of 10^–5^ mE_*h*_/Bohr for the projected FOD gradient is about 100 times the cost of a NIFLOSIC calculation.

## Summary and Outlook

The NIFLOSIC method provides a computationally efficient alternative to traditional FLOSIC by eliminating the need for iterative relaxation of Fermi orbital descriptors. The method is shown to produce localized orbitals that are slightly more localized than SCDM-g orbitals, and also to generate FODs in a single, noniterative self-starting step after DFT calculations. Fully relaxing the density to minimize the PZ energy functional in a generalized KS scheme provides self-interaction corrected density and orbitals. NIFLOSIC yields results that closely reproduce fully self-consistent FLOSIC calculations while remarkably reducing the computational cost. The method can also be utilized to produce initial FODs and densities for FLOSIC calculations. One weakness of the method is that the total electronic energy may be unsuitable for thermochemistry evaluation. However, benchmark tests across diverse molecular systems demonstrate that NIFLOSIC significantly improves HOMO energies and dipole moments, positioning it as a practical and scalable approach for large-scale electronic structure applications where self-interaction correction is essential.

This work offers a method to derive FODs and FLOs directly from KS orbitals, eliminating the FOD optimization and potentially restoring DFT-like computational scaling. Although our results discourage the use of this method with current density functionals for thermochemistry purposes, looking into the future, the resulting computational efficiency encourages the derivation of new explicitly self-interaction-corrected density functionals that are insensitive to the localization scheme, rather than appending new terms onto density-functionals that were developed to be used without self-interaction corrections.
